# Supplementary material for: Environmental DNA from multiple pathogens is elevated near active Atlantic salmon farms
Source: Proc Biol Sci. 2020 Oct 21;287(1937):20202010. doi: 10.1098/rspb.2020.2010 (PMC7661312; doi:10.1098/rspb.2020.2010)
Supplement: A comprehensive description of Methods and Supporting Figures and Tables [file rspb20202010supp1.pdf]

Supplement to: Environmental DNA from multiple pathogens is elevated near active Atlantic salmon farms
doi: 10.1098/rspb.2020.2010

Dylan Shea[1], Andrew Bateman[1,2,3], Shaorong Li[4], Amy Tabata[4], Angela Schulze[4], Gideon Mordecai[5], Lindsey Ogston[1], John P. Volpe[6], L. Neil Frazer[7], Brendan Connors[8], Kristina M. Miller[4], Steven Short[1,9*], Martin Krkošek [1,2*]

[1] Department of Ecology and Evolutionary Biology, University of Toronto, ON
[2] Salmon Coast Field Station, Simoom Sound, BC
[3] Pacific Salmon Foundation, Vancouver, BC
[4] Pacific Biological Station, Fisheries and Oceans Canada, Nanaimo, BC
[5] Department of Medicine, UBC, Vancouver, BC
[6] School of Environmental Studies, University of Victoria, Victoria, BC
[7] Department of Earth Sciences, University of Hawaii at Mānoa, Honolulu, HI
[8] Institute of Ocean Sciences, Fisheries and Oceans Canada, Sidney, BC
[9] Department of Biology, University of Toronto Mississauga, Mississauga ON
[*] Shared last authors

Supplementary Methods:

1) Sample Collection

1.1) Seawater Collection:

At each site we collected a 15 L sample of seawater made up of three 5 L subsamples collected approximately 20 m away from each of three edges of a salmon farm at a depth of two metres using depth-sampling Niskin bottles (General Oceanic, Miami, FL, USA). This depth was chosen as one commonly inhabited by outmigrating juvenile Pacific salmon, and the three sides of each farm were sampled to account for variation in currents among sites at the times of sampling. Due to the fact that most farm tenures are located nearby to the adjacent shoreline, we did not collect samples from the side of each salmon farm closest to shore. At each of these three locations, we also recorded GPS coordinates using a handheld unit (Standard Horizon, Cypress, CA, USA), temperature and salinity using a submersible probe (YSI Incorporated, Yellow Springs, OH, USA), and turbidity

using a white disc (readings taken as depth at which the disc was obscured;[1]). For statistical analyses, temperature, salinity, and turbidity were averaged across the three sub-sampling locations at each farm tenure. We did not sample between a farm and the nearest shoreline where crew accommodations are typically situated. For inactive sites where no farm structure was present, we collected seawater from three locations within the boundaries of the farm tenure.

1.2) Seawater Filtration:

Retention of Cellular (> 0.22μm) Organisms

We pressure filtered seawater samples at 517 mm Hg through 142 mm diameter 0.22 μm pore-size polyethersulfone (PES) filter. During the first step of the filtration process, we loaded each pooled seawater collection into an eleven-litre dispensing pressure vessel (EMD Millipore Corporation, Darmstadt, Germany) and filtered the seawater through a 0.6 μm pore-size (142mm diameter) borosilicate filter (GE Life Sciences, NY, USA) followed by a 0.22 μm Millipore Express Plus polyethersulfone filter (EMD Millipore Corporation, Darmstadt) atop filter stands (EMD Millipore Corporation, Darmstadt, Germany, PN-YY3014236) connected in series. We terminated filtration once we reached our season-standardized water volume between ten and twelve litres. We terminated filtration once we reached our target water volume, standardized within each sampling year, between ten and twelve litres. Using forceps cleaned with ethanol, we placed filters from this initial filtration step in 10 mL cryogenic vials (VWR Scientific, PA, USA) before flash freezing them in liquid nitrogen and storing them at -80°C.

Retention of Sub-cellular *(> 0.22μm) Organisms*

To capture the viral community, we chemically flocculated the filtrate from the first filtration step via the addition of iron chloride (0.018 M). Following a one hour incubation with iron flocculant, we passed the $FeCl_3$-treated filtrate through a 1.0 μm Nucleopore polycarbonate filter membrane (GE Healthcare Life Sciences, Buckinghamshire) supported by a 0.8 μm polyethersulfone filter (Pall Corporation, New York, USA). We processed 9-10 litres of $FeCl_3$-treated filtrate seawater through each polycarbonate filter on top of a support filter. We collected these polycarbonate filters containing iron-flocculated sub-cellular biological material and transferred them to 50 mL centrifuge tubes (VWR Scientific, PA, USA) with ethanol-cleaned forceps, and then stored the tubes at 4°C prior to further processing. Between each filtration step, we rinsed the filter apparatus with approximately 1 L distilled water and wiped filter holders with 70% ethanol. To avoid cross-contamination among sites, we primed the apparatus without filters in place, with approximately 1 L of sample from the focal site before proceeding to retain cellular or viral material.

2) Molecular Sample Processing

2.1) Concentration of Viral (< 0.22μm) Fraction

Viruses were released from their flocculated state and released from filters via the addition of a reducing ascorbic acid buffer (1.51 g TRIS, 3.72 g Na_2-EDTA, 4.07 g $MgCl_2$, 3.52 g ascorbic acid, and nanopure water up to 100 mL). Ascorbic acid buffer was added to Falcon tubes containing viral filters (1 mL buffer/mL $FeCl_3$) and tubes were

left to rinse on a rotator overnight at 4°C. Following rinsing, viruses were concentrated from the solution via ultracentrifugation at 32,000 rpm for three hours at 4°C on top of a 1.5 mL sucrose cushion (PN-L8-70M, Beckman Coulter Inc., Brea, CA, USA). Following ultracentrifugation, the supernatant was removed, and viruses were re-suspended in 280 μL of TE buffer. Samples were transferred to 2 mL tubes and stored at -80°C in preparation for RNA and DNA extraction.

2.2) Nucleic Acid Extraction from Cellular (> 0.22μm) Sample Fraction

Cellular cTAB DNA Extraction:

We concentrated and extracted nucleic acids from cellular (> 0.22 μm) and subcellular (< 0.22 μm) collection filters in preparation for molecular quantification. We extracted and combined total nucleic acids from one half (~ 79 cm^2) of each cellular (> 0.22 μm) filter using a Cetyl trimethylammonium bromide (CTAB)-based extraction protocol, which was optimized for DNA isolation but allowed for the co-precipitation of RNA [2] (Fig. S1). Samples were shipped to the University of Toronto, Mississauga to extract total genetic material from preserved filters. Half of each cellular filter was excised, cut into small fragments to increase exposed surface area, and placed into a 15 mL centrifuge tube (VWR Scientific, PA, USA). A hexadecyltrimethylammonium bromide (CTAB)-based extraction protocol was used to extract total cellular DNA from cellular (0.6 μm and 0.22 μm) filters [3]. Briefly, 15 mL of CTAB buffer (1.5 mL 1M TRIS, 4.2 mL 5M NaCl, 0.6 mL 0.5M EDTA, 8.7 mL nuclease-free water, 75 μL β-mercaptoethanol, 0.6 g polyvinylpyrrolidine, and 0.3 g CTAB powder) was prepared

prior to each extraction. To each extraction tube, we added strips of sample filter, 500 μL CTAB buffer, and 0.25 g each of 213-300 μm and 425-600 μm sterile glass beads. Cells were disrupted via six minutes in a Mini-Beadbeater-96 (PN1001, Biospec, Bartlesville, OK, USA), followed by a one-hour incubation at 55°C. To each tube, 500 μL of a 24:1 Chloroform: Isoamyl alcohol mixture was added and tubes were centrifuged at 21,000xg for 10 minutes and the supernatant was transferred to a new tube. Volumes of all samples were measured with a pipette and 0.08 volumes of 7.5 M ammonium acetate followed by 0.54 volumes of cold (-20°C) isopropanol were added. Samples were inverted to mix and incubated at -20°C for one hour. Extraction tubes were again inverted to mix and centrifuged at 21,000xg for three minutes. The supernatant was removed and 700 μL of cold (-20°C) 70% ethanol was added to each tube and samples were mixed by inverting, followed by centrifugation at 21,000xg for one minute. The supernatant was removed, using care to leave behind pelleted material and 700 μL of cold (-20°C) 95% ethanol and samples were inverted followed by centrifugation for one minute at 21,000xg. The supernatant was again removed without disturbing pelleted material and tubes were spun once again for 1 minute at 21,000xg. Residual supernatant was removed using a micropipette and samples were spun again at 21,000xg to pellet cellular DNA. Samples were left open in the clean hood for thirty minutes to facilitate the evaporation of any remaining ethanol and 25 μL of Tris-EDTA (TE) was added to each. All tubes were incubated for one hour at 55°C to allow pelleted nucleic acids to return to solution and the four tubes from each sample were combined. Extractions from 1.6 μm and 0.22 μm filters were combined for each sample and were stored at -20°C prior to downstream analysis.

2.3) Nucleic Acid Extraction of Concentrated Viral (< 0.22μm) Sample Fraction

Total RNA and DNA were extracted from concentrated viral samples using the QIAmp viral RNA mini kit and eluted in 80 μL of AVE buffer (Qiagen, CA, USA). We performed the optional double elution step (40 μL + 40 μL) as well as one optional spin to remove residual buffer AW2. Of this extraction, 40 μL was reserved for downstream analysis of DNA viruses while the remaining 40 μL was utilized as template for complementary DNA (cDNA) synthesis to facilitate the quantification of RNA viruses.

2.4) cDNA synthesis from viral RNA

We synthesized complementary DNA (cDNA) from RNA template recovered during nucleic acid extractions of both cellular (> 0.22μm; RNA recovered during CTAB DNA extraction) and sub-cellular (< 0.22μm; RNA extracted from concentrated free-virus fraction using QIAmp viral RNA mini kit) filter fractions from each site. We tested both of these sample fractions in order to facilitate the quantification of the RNA viruses of interest in both cell-associated and cell-free states. First strand synthesis was performed by combining 16 μL of viral RNA template with 4 μL of SuperScript VILO cDNA master mix (ThermoFisher, Carlsbad, CA, USA). Samples were placed in a BioRad C1000 thermocycler (BioRad Laboratories, Hercules, CA, USA) and incubated according to the manufacturer's instructions: (25°C for 10 min, 42°C for 60 min, 85°C for 5 min). Positive controls, containing known RNA template, and negative controls, containing RNA template without Reverse-Transcriptase enzyme, were synthesized simultaneously

with environmental RNA samples. After cDNA synthesis, samples were centrifuged and stored at -20°C prior to downstream quantification.

3) ***Specific Target Amplification (STA) and Quantitative PCR (qPCR)***

3.1) STA on Extracted Samples

In order to increase the sensitivity of qPCR reactions on the microfluidics Fluidigm BioMark HD platform, which occur on a chip containing small 7 nL reaction wells, samples underwent an initial PCR-based enrichment, following Fluidigm BioMark recommendations. This is to account for the >1000-fold difference in sample volume between conventional qPCR (25 μL) and Biomark dynamic array (7 nL) reactions. Samples were pre-amplified in their three independent fractions (cellular DNA, viral DNA, and viral cDNA) prior to qPCR. The samples were pre-amplified using the TaqMan PreAmp Master Mix, and then treated with exonuclease enzyme to digest residual primers before proceeding with qPCR.

We prepared a primer mix, containing all assay primer pairs for a final concentration of 200 nM of each forward and reverse primer (Table S3). We pre-amplified samples in five microlitre STA reactions on sealed assay plates containing 1.25 μL primer mix, 1.25 μL template DNA, and 2.5 μL TaqMan PreAmp Master Mix (Applied Biosystems, CA, USA) under the following cycling conditions: 95°C for 10 min followed by fourteen cycles of: (95°C for 15 min, 60°C for 4 min). Following pre-amplification, we treated STA reactions with ExoSAP-IT exonuclease enzyme (Affymetrix, CA) under the reaction conditions: (37°C for 15 min, 80°C for 15 min) to digest residual primers. After primer digestion, we diluted STA samples 5-fold in DNA

suspension buffer (TEKnova) and stored them at -20°C in preparation for quantification on the BioMark Platform.

3.2) BioMark Microfluidics Quantitative PCR (qPCR):

We quantified the pre-amplified samples using the 96.96 Dynamic Array^{TM} run on the BioMark^{TM} HD microfluidics qPCR platform (Fluidigm Corporation, CA, USA). High throughput Fluidigm chips allowed us to simultaneously screen samples for all microparasite species as well as for Atlantic salmon eDNA in duplicate (Table S2; modified from [4]). The amplification procedures followed the protocol in [4]. We pre-amplified and quantified cellular and sub-cellular fractions on separately and additionally analyzed sub-cellular cDNA (synthesized from extracted RNA) separately from sub-cellular DNA, resulting in three distinct molecular analysis streams (Fig. S1). We consolidated sample fractions into two molecular analysis streams prior to qPCR during 2016 surveys (See Table S2 for a description of sample fraction-specific quantification procedure employed in 2016). We incorporated control samples for each step of sample extraction, amplification, and quantification, which were carried forward and assessed alongside experimental samples on the BioMark platform.

Across the three surveys, we screened the samples for a total of 39 microparasite species as well as Atlantic salmon eDNA in duplicate using published Taqman assays (Table S3; modified from [4]). Of the 39 microparasites for which we assessed seawater samples, 24 were surveyed across all three sampling years, 11 were unique to 2016 surveys and 3 were unique to 2017/2018 surveys (Table S3). The microparasites we selected for surveillance had been detected in a previous monitoring program comprising

>28,000 cultured and all five wild salmonid species in British Columbia [5]. Assays used for this study were designed to quantify 15 eukaryotes, 12 bacteria, and 12 viruses known or suspected to be pathogenic in salmon, some causing acute disease and others more opportunistic, largely causing disease in stressed fish.

We prepared samples for quantification according to the protocols outlined in Miller et al. (2016). Briefly, we prepared a 5 μL sample mix for each sample, containing: 1X TaqMan Universal Master Mix (Applied Biosystems, PN 4369016), 1X GE Sample Loading Reagent (Fluidigm, PN 85000746), and 2.25 μL of ExoSAP-treated STA sample. Additionally, we prepared a 5 μL assay mix for each respective TaqMan qPCR assay, containing 9 μM of each forward and reverse primer, 2 μM each of FAM-MGB and NED-MGB fluorescent probes (Applied Biosystems, Foster City, CA, USA), and 1X Assay Loading Reagent (Fluidigm, PN 85000736).

We carried out reactions using the GE 96X96 TaqMan qPCR program with a hot start followed by forty cycles of: 95 °C for fifteen seconds followed by 60 °C for one minute (Fluidigm Corporation, CA, USA). We analyzed results using real-time PCR software (Fluidigm Corporation, CA, USA) and the number of elapsed qPCR cycles before each sample reached a threshold level of fluorescence (Ct) was recorded. We generated microparasite detection results from the qPCR outputs of multiple separate Fluidigm chips, which also represented independent STA reactions. Standards and controls (outlined below) were nearly identical among distinct chips and STA’s, suggesting that it was appropriate to analyze the data from multiple chips within each season together.

3.3) BioMark Controls:

We incorporated a number of control samples throughout the various stages of sample processing and amplification, all of which were analyzed on Fluidigm chips along with experimental samples. Below, we describe the various controls we incorporated for sample extraction and processing, specific target amplification (STA), and Fluidigm chip controls. We included nucleic acid extraction negative controls which consisted of replicate milliQ water samples, which had been taken through the various steps of the cellular nucleic acid extraction procedure to ensure none of the reagents used during the extraction of experimental samples contained contaminating nucleic acids. This same procedure was repeated for the viral extraction procedure. These extraction controls were subjected to cDNA synthesis, STA'd, and run on Fluidigm chips in parallel with experimental samples. Negative controls did not amplify with any of the TaqMan assays, indicating that extraction reagents were free of contaminating template DNA.

For cDNA controls we included samples positive for a number of the target RNA viruses, in the same cDNA run as experimental samples with and without Reverse Transcriptase to ensure that cDNA synthesis proceeded successfully (positive control) and that no cDNA was detected in the absence of RT (no-RT) or in the absence of RNA template (no-template). For the analysis of 2016 samples, this cDNA control sample consisted of pooled RNA extracted from Atlantic salmon that were positive for a number of the target RNA viruses. For 2017 and 2018 molecular analysis, this cDNA control consisted of pooled RNA extracted from Sockeye salmon that were similarly confirmed to be positive for a number of the target RNA viruses included on the panel. In both cases, we observed no amplification of assays targeting RNA-based organisms in our no-

RT negative control, indicating that that cDNA reagents were free of contaminating cDNA and that the RNA-based assays were specific to RNA-based agents and did not cross react with DNA template. Additionally, we did not observe amplification in our no-template controls, further indicating that the reagents used for cDNA synthesis were free of contaminating template. We also included a cDNA positive control, consisting of our pooled positive RNA, VILO cDNA master mix, and Reverse Transcriptase enzyme. In all years, we observed RNA virus amplification of these positive control samples, indicating that the cDNA reaction was successful in generating DNA copies of the RNA template contained within these samples. This cDNA positive control was carried forward to serve as an STA control.

We included a number of controls for the specific target amplification (STA) reaction prior to quantification. STA positive control consisted of template from our cDNA positive control, primers, and STA master mix and was STA'd along with experimental samples. This STA positive was subsequently run on the Fluidigm chip alongside the cDNA positive control, which had not been STA'd (no-STA positive) to confirm that the STA reaction was successful in enriching samples by ~ 1000-fold. On all chips, we observed a Ct-difference of approximately 10 cycles (STA pos ~ no-STA pos +10) for all assays that were positive in these two samples, indicating that the STA reaction achieved the intended enrichment. STA negative control samples consisted of STA master mix with water instead of template to ensure STA reagents were free of contaminating template. In all STA reactions, we observed no amplification of STA negative controls indicating STA reagents were free of contaminating template. In addition to all of the previously described positive and negative controls, which were

carried forward and run on Fluidigm chips, we included Fluidigm chip negative controls, which consisted of sample wells which received water instead of template DNA. We observed no amplification in chip negative controls. For chip positive controls, all reaction wells contained ROX fluorescent dye to confirm that all wells contained the same amount of master mix.

Artificial construct positive control samples (APC clones), which are comprised of cloned and precisely quantitated synthesized amplicons for each assay were combined for all assays and serially diluted to facilitate assessments of assay efficiency and copy number calculations from samples. Each APC contained an extra sequence for which a second fluorescent probe, the NED-MGB probes, were derived and included in each sample assay to track any potential contamination of high concentration controls in our samples. Serial dilutions of combined APC standards were loaded onto the reaction plate last, immediately prior to amplification to limit the likelihood of contamination. Once we had prepared both assay and sample plates, we loaded each into its respective well on a 96.96 dynamic array chip and transferred the array to the BioMark HD instrument. We observed no indications that cross-contamination between samples and standards had occurred; however, we excluded all FAM fluorescence detections for which any non-target NED fluorescence was also detected.

Supplementary Figures and Tables

Supplementary Table 1. Coordinates, collection dates, and site details for all sampled farm tenures. Site numbers correspond to the point labels on map (Figure 1) depicting the geographic location of sampling sites.

			2016		*2017*		*2018*	
**Site**	**Latitude**	**Longitude**	**Date**	**Status**	**Date**	**Status**	**Date**	**Status**
1	50.879	-126.902	2016-03-28	Inactive			2018-07-19	Inactive
2	50.865	-126.922	2016-03-28	Inactive			2018-05-29	Inactive
3	50.854	-126.759	2016-03-29	Inactive	2017-08-08	Active	2018-07-19	Active
4	50.851	-126.717	2016-03-29	Active			2018-07-19	Inactive
5	50.838	-126.664	2016-03-29	Inactive			2018-07-20	Active
6	50.786	-126.686	2016-03-31	Inactive	2017-08-15	Active	2018-07-12	Active
7	50.831	-126.598	2016-03-29	Active	2017-08-08	Active	2018-07-20	Active
8	50.71	-126.662	2016-03-30	Inactive			2018-07-12	Inactive
9	50.745	-126.613	2016-03-31	Inactive				
10	50.835	-126.497	2016-03-31	Inactive			2018-07-20	Inactive
11	50.619	-126.705	2016-04-01	Active	2017-08-03	Active	2018-07-14	Active
12	50.657	-126.666	2016-04-01	Active	2017-08-14	Inactive		
13	50.797	-126.495	2016-03-31	Active	2017-08-08	Active	2018-05-15	Active
14	50.722	-126.569	2016-03-30	Active	2017-08-15	Inactive	2018-07-12	Inactive
15	50.649	-126.618	2016-04-01	Active	2017-08-14	Active	2018-07-14	Inactive
16	50.602	-126.633	2016-04-01	Inactive			2018-05-25	Active
17	50.809	-126.414	2016-03-28	Inactive			2018-05-28	Inactive
18	50.848	-126.319	2016-03-28	Active	2017-08-07	Active	2018-07-11	Active
19	50.671	-126.476	2016-03-30	Active	2017-08-15	Inactive	2018-05-31	Active
20	50.628	-126.479	2016-03-30	Inactive				
21	50.608	-126.363	2016-04-02	Inactive			2018-07-10	Active
22	50.698	-126.257	2016-04-02	Active	2017-08-07	Active	2018-07-11	Active
23	50.601	-126.348	2016-04-01	Inactive			2018-06-02	Active
24	50.654	-126.29	2016-04-02	Active	2017-08-06	Active	2018-07-10	Active
25	50.612	-126.332	2016-04-02	Inactive			2018-07-01	Inactive
26	50.673	-126.186	2016-04-02	Active	2017-08-06	Active	2018-07-13	Active
27	50.446	-125.97	2016-03-17	Inactive				
28	50.488	-125.889	2016-03-18	Inactive	2017-08-04	Inactive	2018-07-09	Active
29	50.475	-125.809	2016-03-18	Inactive	2017-08-03	Active	2018-07-05	Inactive
30	50.415	-125.768	2016-03-17	Active	2017-08-03	Active	2018-07-09	Inactive
31	50.41	-125.7	2016-03-17	Inactive	2017-08-03	Inactive		
32	50.415	-125.66	2016-03-17	Active	2017-08-04	Inactive	2018-07-09	Active
33	50.388	-125.528	2016-03-18	Inactive			2018-07-09	Inactive
34	50.453	-125.396	2016-03-16	Inactive	2017-08-02	Inactive	2018-07-09	Inactive

35	50.488	-125.357	2016-03-16	Inactive	2017-08-05	Inactive	2018-07-08	Active
36	50.486	-125.276	2016-03-15	Inactive			2018-07-08	Inactive
37	50.46	-125.296	2016-03-15	Inactive			2018-07-08	Inactive
38	50.372	-125.38	2016-03-19	Inactive	2017-08-03	Inactive	2018-07-07	Inactive
39	50.41	-125.34	2016-03-16	Inactive			2018-07-08	Inactive
40	50.486	-125.249	2016-03-15	Inactive			2018-07-08	Inactive
41	50.426	-125.306	2016-03-16	Active	2017-08-03	Active	2018-07-08	Active
42	50.47	-125.26	2016-03-15	Inactive			2018-07-08	Inactive
43	50.35	-125.343	2016-03-19	Inactive			2018-07-07	Inactive
44	50.303	-125.337	2016-03-19	Active	2017-08-01	Active	2018-07-03	Active
45	50.286	-125.349	2016-03-18	Active			2018-07-05	Active
46	50.31	-125.316	2016-03-19	Active			2018-07-03	Active
47	50.324	-125.261	2016-03-20	Active	2017-08-01	Active	2018-07-05	Active
48	50.133	-125.333	2016-03-22	Inactive			2018-07-03	Active
49	50.254	-125.212	2016-03-20	Inactive			2018-07-05	Inactive
50	50.342	-125.072	2016-03-14	Inactive			2018-07-07	Inactive
51	50.092	-125.313					2018-07-03	Inactive
52	50.181	-125.15	2016-03-22	Inactive			2018-07-06	Inactive
53	50.321	-125.01	2016-03-14	Active			2018-07-07	Active
54	50.189	-125.142	2016-03-22	Inactive			2018-07-05	Inactive
55	50.152	-125.147	2016-03-11	Inactive			2018-07-06	Inactive
56	50.145	-125.152	2016-03-11	Inactive			2018-07-06	Inactive
57	50.251	-124.819	2016-03-22	Inactive			2018-07-04	Inactive
58	50.29	-124.636	2016-03-22	Inactive			2018-07-04	Inactive

*Active sites were farming Atlantic salmon at the time of sampling.

*Filtration volumes one and two represent the volume of water processed during the initial, pre-filtration step and the volume of water processed during the secondary, viral filtration step, respectively.

Supplementary Figure 1. A schematic depicting the generalized workflow from sample collection to molecular analysis.

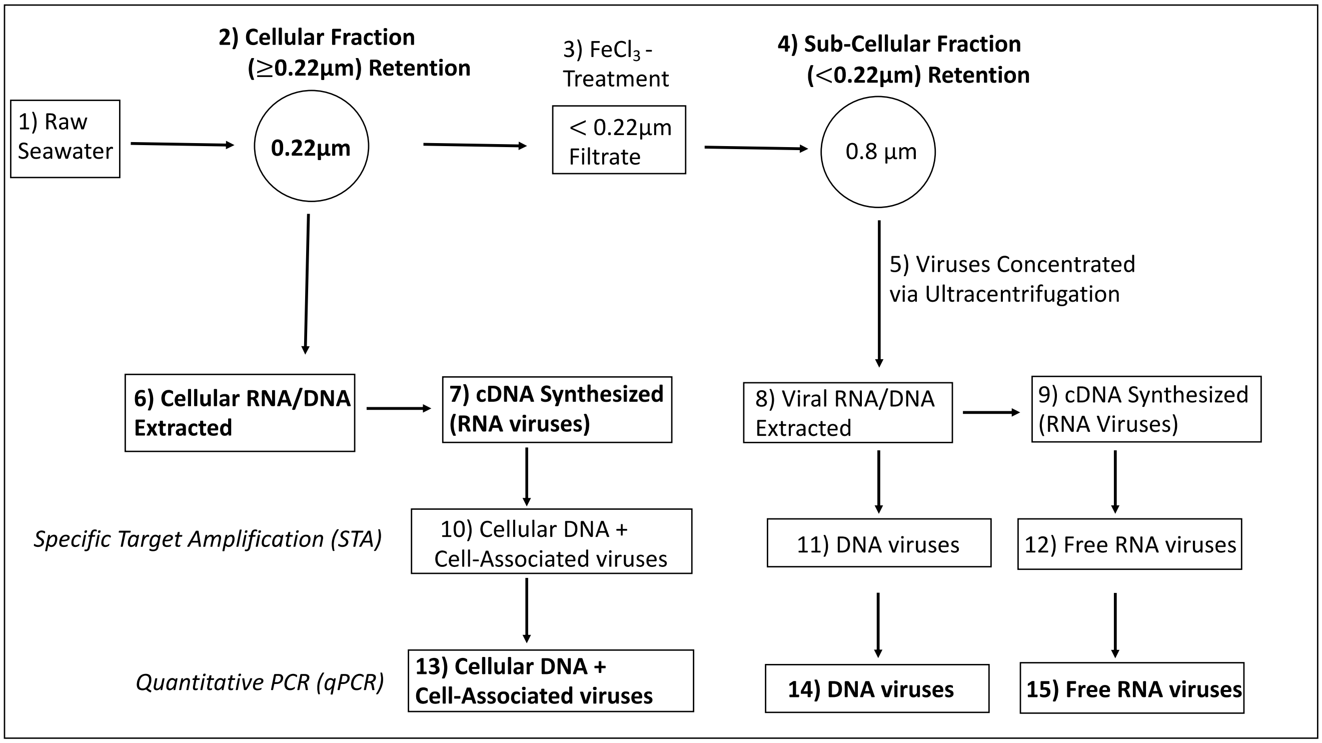

*****Steps that are bolded were subject to minor inter-annual methodological variations.
*See Table S3 for a brief description of interannual methodological changes and for a more detailed description and rationale for specific changes.

Supplementary Table 2. Methodological variation between 2016, 2017, and 2018 seawater surveys and rationales for each change.

**Step**		**Variation**	**Rationale**
Sites sampled		2016 (57), 2017 (24), 2018 (53)	-Opportunistic sampling
Filtration of cellular material (≥0.22µm)	Volume	2016: 10.10$\pm$ 0.36 ($\bar{x} \pm SD$); 2017: 14.65$\pm$ 1.82 ($\bar{x} \pm SD$); 2018: 11.36$\pm$ 0.55 ($\bar{x} \pm SD$)	-Maximize processed volumes to increase cellular sample concentration
Cellular RNA/DNA Extraction	Filters used to capture cellular microbes	2016; 2017: 0.6µm GF + 0.22µm PEF<br>2018: 0.22 µm PEF	-Increase nucleic acid concentration per filter surface area
Filtration of sub-cellular material (<0.22µm)	Volume	2016: 9.09$\pm$ 0.24 ($\bar{x} \pm SD$); 2017: 10.99$\pm$ 2.19 ($\bar{x} \pm SD$); 2018: 9.37$\pm$ 0.40 ($\bar{x} \pm SD$)	-Maximize processed volumes to increase concentration of sub-cellular material
	Portion of filter analyzed	2016: one quarter (~39.5 cm^2)<br>2017/2018: one half (~79 cm^2)	-Increase concentration of nucleic acids recovered from cellular filters
Cellular cDNA Synthesis	Quantify cell-associated RNA viruses	2016: did not synthesize cDNA from cellular RNA<br>2017/2018: synthesized cDNA from cellular RNA to facilitate quantification of cell-associated RNA viruses	-Quantify viruses in both "free-virus" and "cell-associated" states
Assay for Atlantic salmon DNA		Atlantic salmon qPCR assay used in 2016 differed from one used on 2017/2018 samples	-Utilized most current validated assay
Eukaryotic and bacterial species		2016: assessed 9 Eukaryotic and 2 Bacterial microparasites that were not assessed in 2017/2018.	-Updated our pathogen panel to exclude freshwater microparasites
DNA viruses	Pooling of cellular and subcellular material	number and composition of qPCR sample fractions run separately:<br>2016 (2: [Cell DNA + Sub. Cell DNA], [Sub Cell RNA]),<br>2017; 2018 (3: [Cell DNA], [Sub. Cell DNA], [Sub Cell RNA])	-Minimize dilution of viral DNA to increase assay sensitivity
	Species assayed	In 2017/2018, we assessed 1 DNA viruses that were not included in 2016 surveys.	-Updated pathogen panel with most current list of known microparasites
RNA viruses		In 2017/2018, we assessed 2 RNA viruses that were not included in 2016 surveys.	-Updated pathogen panel with most current list of known microparasites

Supplementary Table 3. Target species that were included in 2016, 2017, and 2018 surveys.

Agent	Classification	Organism	Survey Year	Ref	Forward Primer Sequence (5'-3')<br>Reverse Primer Sequence (5'-3')<br>Probe Sequence (FAM-5'-3'-MGB-NFP)
**Eukaryote**	Chordata	*Salmo salar (Atlantic salmon)*	2017; 2018	[6]	F: CGCCCTAAGTCTCTTGATTCGA<br>R: CGTTATAAATTTGGTCATCTCCCAGA<br>P: AGAACTCAGCCAGCCTG
**Eukaryote**	Chordata	*Salmo salar (Atlantic salmon)*	2016	[7]	F: AGCAGAACTCAGCCAGCCT<br>R: AAAGGAGGGAGGGAGAAGTCAA<br>P: CCTTCTGGGAGATGACC
**Eukaryote**	Choanozoa	*Sphaerothecum destructuens*	2016	[4]*	F: GGGTATCCTTCCTCTCGAAATTG<br>R: CCCAAACTCGACGCACACT<br>P: CGTGTGCGCTTAAT
**Eukaryote**	Choanozoa	*Ichthyophonus hoferi*	2016	[8]*	F: GTCTGTACTGGTACGGCAGTTTC<br>R: TCCCGAACTCAGTAGACACTCAA<br>P: TAAGAGCACCCACTGCCTTCGAGAAGA
**Eukaryote**	Platyhelminthes	*Nanophyetus salmincola*	2016	[4]*	F: CGATCTGCATTTGGTTCTGTAACA<br>R: CCAACGCCACAATGATAGCTATAC<br>P: TGAGGCGTGTTTTATG
**Eukaryote**	Eudiscosea	*Neoparamoeba perurans*	2016	[9]*	F: GTTCTTTCGGGAGCTGGGAG<br>R: GAACTATCGCCGGCACAAAAG<br>P: CAATGCCATTCTTTTCGGA
**Eukaryote**	Ciliophora	*Ichthyophthirius multifiliis*	2016	[4]*	F: AAATGGGCATACGTTTGCAAA<br>R: AACCTGCCTGAAACACTCTAATTTTT<br>P: ACTCGGCCTTCACTGGTTCGACTTGG
**Eukaryote**	Myxozoa	*Myxobolus arcticus*	2016	[4]*	F: TGGTAGATACTGAATATCCGGGTTT<br>R: AACTGCGCGGTCAAAGTTG<br>P: CGTTGATTGTGAGGTTGG

Supplementary Table 3. Cont'd

Agent	Classification	Organism	Survey Year	Ref	Forward Primer Sequence (5'-3')<br>Reverse Primer Sequence (5'-3')<br>Probe Sequence (FAM-5'-3'-MGB)
**Eukaryote**	Myxozoa	*Myxobolus insidiosus*	2016	[4]*	F: CCAATTTGGGAGCGTCAAA<br>R: CGATCGGCAAAGTTATCTAGATTCA<br>P: CTCTCAAGGCATTTAT
**Eukaryote**	Myxozoa	*Kudoa thyrsites*	2016; 2017; 2018	[10]*	F: TGGCGGCCAAATCTAGGTT<br>R: GACCGCACACAAGAAGTTAATCC<br>P: TATCGCGAGAGCCGC
**Eukaryote**	Myxozoa	*Parvicapsula pseudobranchicola*	2016; 2017; 2018	[11]*	F: CAGCTCCAGTAGTGTATTTCA<br>R: TTGAGCACTCTGCTTTATTCAA<br>P: CGTATTGCTGTCTTTGACATGCAGT
**Eukaryote**	Myxozoa	*Parvicapsula kabatai*	2016; 2017; 2018	[4]*	F: CGACCATCTGCACGGTACTG<br>R: ACACCACAACTCTGCCTTCCA<br>P: CTTCGGGTAGGTCCGG
**Eukaryote**	Myxozoa	*Parvicapsula minibicornis*	2016; 2017; 2018	[12]*	F: AATAGTTGTTTGTCGTGCACTCTGT<br>R: CCGATAGGCTATCCAGTACCTAGTAAG<br>P: TGTCCACCTAGTAAGGC
**Eukaryote**	Microsporidia	*Facilispora margolisi*	2016; 2017; 2018	[4]*	F: AGGAAGGAGCACGCAAGAAC<br>R: CGCGTGCAGCCCAGTAC<br>P: TCAGTGATGCCCTCAGA
**Eukaryote**	Microsporidia	*Loma salmonae*	2016	[4]*	F: GGAGTCGCAGCGAAGATAGC<br>R: CTTTTCCTCCCTTTACTCATATGCTT<br>P: TGCCTGAAATCACGAGAGTGAGACTACCC
**Eukaryote**	Microsporidia	*Nucleospora salmonis*	2016	[13]*	F: GCCGCAGATCATTACTAAAAACCT<br>R: CGATCGCCGCATCTAAACA<br>P: CCCCGCGCATCCAGAAATACGC

Supplementary Table 3. Cont'd

Agent	Classification	Organism	Survey Year	Ref	Forward Primer Sequence (5'-3')<br>Reverse Primer Sequence (5'-3')<br>Probe Sequence (FAM-5'-3'-MGB)
**Eukaryote**	Microsporidia	*Desmozoon lepeophtherii*	2016; 2017; 2018	[14]*	F: CGGACAGGGAGCATGGTATAG<br>R: GGTCCAGGTTGGGTCTTGAG<br>P: TTGGCGAAGAATGAAA
**Eukaryote**	Bacteroidetes	*Flavobacterium psychrophilum*	2016	[15]*	F: GATCCTTATTCTCACAGTACCGTCAA<br>R: TGTAAACTGCTTTTGCACAGGAA<br>P: AAACACTCGGTCGTGACC
**Eukaryote**	Bacteroidetes	*Vibrio anguillarum*	2016; 2017; 2018	[4]*	F: CCGTCATGCTATCTAGAGATGTATTTGA<br>R: CCATACGCAGCCAAAAATCA<br>P: TCATTTCGACGAGCGTCTTGTTCAGC
**Eukaryote**	Bacteroidetes	*Vibrio salmonicida*	2016; 2017; 2018	[4]*	F: GTGTGATGACCGTTCCATATTT<br>R: GCTATTGTCATCACTCTGTTTCTT<br>P: TCGCTTCATGTTGTGTAATTAGGAGCGA
**Eukaryote**	Bacteroidetes	*Tenacibaculum maritimum*	2016; 2017; 2018	[16]	F: TGCCTTCTACAGAGGGATAGCC<br>R: CTATCGTTGCCATGGTAAGCCG<br>P: CACTTTGGAATGGCATCG
**Eukaryote**	Bacteroidetes	*Yersinia ruckeri*	2016; 2017; 2018	[17]*	F: TCCAGCACCAAATACGAAGG<br>R: ACATGGCAGAACGCAGAT<br>P: AAGGCGGTTACTTCCCGGTTCCC
**Eukaryote**	Chlamydiae	*Piscichlamydia salmonis*	2016; 2017; 2018	[18]*	F: TCACCCCCAGGCTGCTT<br>R: GAATTCCATTTCCCCCTCTTG<br>P: CAAAACTGCTAGACTAGAGT
**Eukaryote**	Chlamydiae	*Cand. Syngnamydia salmonis*	2016; 2017; 2018	[19]*	F: GGGTAGCCCGATATCTTCAAAGT<br>R: CCCATGAGCCGCTCTCTCT<br>P: TCCTTCGGGACCTTAC

Supplementary Table 3. Cont'd

Agent	Classification	Organism	Survey Year	Ref	Forward Primer Sequence (5'-3')<br>Reverse Primer Sequence (5'-3')<br>Probe Sequence (FAM-5'-3'-MGB)
**Bacterium**	Proteobacteria	*Aeromonas salmonicida*	2016; 2017; 2018	[20]*	F: TAAAGCACTGTCTGTTACC<br>R: GCTACTTCACCCTGATTGG<br>P: ACATCAGCAGGCTTCAGAGTCACTG
**Bacterium**	Proteobacteria	*Piscirickettsia salmonis*	2016; 2017; 2018	[21]*	F: TCTGGGAAGTGTGGCGATAGA<br>R: TCCCGACCTACTCTTGTTTCATC<br>P: TGATAGCCCCGTACACGAAACGGCATA
**Bacterium**	Proteobacteria	*Moritella viscosa*	2016; 2017; 2018	[4]	F: CGTTGCGAATGCAGAGGT<br>R: AGGCATTGCTTGCTGGTTA<br>P: TGCAGGCAAGCCAACTTCGACA
**Bacterium**	Proteobacteria	*Cand. Branchiomonas cysticola*	2016; 2017; 2018	[22]*	F: AATACATCGGAACGTGTCTAGTG<br>R: GCCATCAGCCGCTCATGTG<br>P: CTCGGTCCCAGGCTTTCCTCTCCCA
**Bacterium**	Actinobacteria	*Renibacterium salmoninarum*	2016	[23]*	F: CAACAGGGTGGTTATTCTGCTTTC<br>R: CTATAAGAGCCACCAGCTGCAA<br>P: CTCCAGCGCCGCAGGAGGAC
**Virus**	Group I: dsDNA	*Erythrocytic necrosis virus*	2016; 2017; 2018	[24]*	F: CGTAGGGCCCCAATAGTTTCT<br>R: GGAGGAAATGCAGACAAGATTTG<br>P: TCTTGCCGTTATTTCCAGCACCCG
**Virus**	Group II: ssDNA	*Pacific salmon parvovirus*	2017; 2018	[4]*	F: CCCTCAGGCTCCGATTTTTAT<br>R: CGAAGACAACATGGAGGTGACA<br>P: CAATTGGAGGCAACTGTA
**Virus**	Group III:dsRNA	*Piscine orthoreovirus*	2016; 2017; 2018	[25]*	F: TGCTAACACTCCAGGAGTCATTG<br>R: TGAATCCGCTGCAGATGAGTA<br>P: CGCCGGTAGCTCT

Supplementary Table 3. Cont'd

Agent	Classification	Organism	Survey Year	Ref	Forward Primer Sequence (5'-3')<br>Reverse Primer Sequence (5'-3')<br>Probe Sequence (FAM-5'-3'-MGB)
**Virus**	Group III: dsRNA	*Putative totivirus*	2016; 2017; 2018	[26]	F: TCTGCGCGCTGCACCTA<br>R: ATGCGGAGGAACTCACACACT<br>P: CAAGTGCTACACTGCG
**Virus**	Group III: dsRNA	*Chinook aquareovirus*	2017; 2018	[27]	F: AACTTTCGGCTTTCTGCTATGC<br>R: GAGGACAAGGGTCTCCATCTGA<br>P: TTAATTGCGGTACTGCTC
**Virus**	Group IV: +ssRNA	*Encephalopathy and retinopathy virus*	2016; 2017; 2018	[28]*	F: TTCCAGCGATACGCTGTTGA<br>R: CACCGCCCGTGTTTGC<br>P: AAATTCAGCCAATGTGCCCC
**Virus**	Group IV: +ssRNA	*Pacific salmon nidovirus*	2016; 2017; 2018	[27]	F: GGATAATCCCAACCGAAAAGTTT<br>R: GCATGAAATGTTGTCTCGGTTTAA<br>P: CGATCCCGATTATC
**Virus**	Group IV: +ssRNA	*Cutthroat trout virus*	2016; 2017; 2018	[26]	F: CCACTTGTCGCTACGATGAAAC<br>R: CGCCTCCTTTGCCTTTCTC<br>P: ATGCCGGGCCATC
**Virus**	Group IV: +ssRNA	*Putative narna-like virus*	2016; 2017; 2018	[26]	F: TGTCCCTGAAGATTCATTTCGA<br>R: CTATGTAAAGCCTCGTCGGTGAT<br>P: TCCTAGGTGATGATATAAT
**Virus**	Group IV: +ssRNA	*Atlantic salmon Calicivirus*	2017; 2018	[26]	F: ACCGACTGCCCGGTTGT<br>R: CTCCGATTGCCTGTGATAATACC<br>P: CTTAGGGTTAAAGCAGTCG

Supplementary Table 3. Cont'd

Agent	Classification	Organism	Survey Year	Ref	Forward Primer Sequence (5'-3')<br>Reverse Primer Sequence (5'-3')<br>Probe Sequence (FAM-5'-3'-MGB)
**Virus**	Group V: -ssRNA	*Salmon pescarenavirus 1*	2016; 2017; 2018	[27]	F: CCTGCCTCTTTGCTCATTGTG<br>R: AGAAAAAGCTGTGGTACTTTAGAAAGC<br>P: ATCCGCCTAACGGTTGG
**Virus**	Group V: -ssRNA	*Salmon pescarenavirus 2*	2016; 2017; 2018	[27]	F: AACATGAAGGGCGATTCGTT<br>R: CAGCCCGCGGACTGAGT<br>P: CAAGTGATGTAAGCTTG<br>P: TCCTAGGTGATGATATAAT

*References to the publication where each assay was initially reported and sequences for primers and probes are reported in subsequent columns.

*Bolded assay references refer to qPCR assays which were designed based on the findings of the referenced work but have not previously been reported.

*Asterisks indicate assays which were evaluated for efficiency, specificity, and sensitivity, during performance assessments [4] across ~ 350,000 qPCR reactions [4].

Supplementary Table 4. Pathogen detections by site status and sampling year.

			2016			2017			2018		
**Agent**	**Classification**	**Organism**	**Total**	**Active**	**Inactive**	**Total**	**Active**	**Inactive**	**Total**	**Active**	**Inactive**
Eukaryote	Chordata	*Salmo salar (Atlantic salmon)*	14 (0.25)	12 (0.6)	2 (0.05)	17 (0.71)	13 (0.87)	4 (0.44)	43 (0.81)	26 (1.0)	17 (0.63)
Eukaryote	Choanozoa	*Ichthyophonus hoferi*	1 (0.02)	1 (0.05)	0 (0)	-	-	-	-	-	-
Eukaryote	Cnidaria	*Kudoa thyrsites*	-	-	-	1 (0.04)	1 (0.07)	0 (0)	1 (0.02)	1 (0.04)	0 (0)
Eukaryote	Cnidaria	*Parvicapsula pseudobranchicola*	ND	ND	ND	1 (0.04)	1 (0.07)	0 (0)	5 (0.09)	3 (0.12)	2 (0.07)
Eukaryote	Cnidaria	*Parvicapsula kabatai*	ND	ND	ND	5 (0.21)	2 (0.13)	3 (0.33)	9 (0.17)	4 (0.15)	5 (0.19)
Eukaryote	Microsporidia	*Facilispora margolisi*	1 (0.02)	1 (0.05)	0 (0)	2 (0.0)	2 (0.13)	0 (0)	12 (0.23)	5 (0.19)	7 (0.26)
Eukaryote	Microsporidia	*Desmozoon lepeophtherii*	12 (0.21)	6 (0.30)	6 (0.16)	22 (0.92)	13 (0.87)	9 (1)	51 (0.96)	26 (1)	25 (0.93)
Prokaryote	Bacteroidetes	*Flavobacterium psychrophilum*	2 (0.04)	1 (0.05)	1 (0.05)	-	-	-	-	-	-
Prokaryote	Bacteroidetes	*Vibrio anguillarum*	1 (0.02)	0 (0)	1 (0.03)	3 (0.13)	3 (0.2)	0 (0)	2 (0.04)	1 (0.04)	1 (0.04)
Prokaryote	Bacteroidetes	*Vibrio salmonicida*	2 (0.04)	1 (0.05)	1 (0.03)	-	-	-	-	-	-
Prokaryote	Bacteroidetes	*Tenacibaculum maritimum*	2 (0.04)	2 (0.10)	0 (0)	3 (0.13)	3 (0.2)	0 (0)	14 (0.26)	13 (0.5)	1 (0.04)
Prokaryote	Bacteroidetes	*Yersinia ruckeri*	2 (0.04)	0 (0)	2 (0.05)	ND	ND	ND	2 (0.04)	2 (0.08)	0 (0)
Prokaryote	Chlamydiae	*Candidatus Syngnamydia salmonis*	52 (0.91)	18 (0.90)	33 (0.89)	23 (0.96)	14 (0.93)	9 (1)	53 (1)	26 (1)	27 (1)
Prokaryote	Proteobacteria	*Piscirickettsia salmonis*	17 (0.30)	11 (0.55)	6 (0.16)	20 (0.83)	13 (0.87)	7 (0.78)	42 (0.79)	23 (0.88)	19 (0.7)
Prokaryote	Proteobacteria	*Moritella viscosa*	10 (0.18)	8 (0.40)	2 (0.05)	ND	ND	ND	3 (0.06)	3 (0.12)	0 (0)
Prokaryote	Proteobacteria	*Candidatus Branchiomonas cysticola*	ND	ND	ND	7 (0.29)	4 (0.27)	3 (0.33)	20 (0.38)	7 (0.27)	13 (0.48)
Virus	Group I: dsDNA	*Erythrocytic necrosis virus*	15 (0.26)	7 (0.35)	8 (0.22)	12 (0.5)	7 (0.47)	5 (0.56)	26 (0.49)	11 (0.42)	15 (0.56)
Virus	Group III: dsRNA	*Piscine reovirus*	ND	ND	ND	1 (0.04)	1 (0.07)	0 (0)	ND	ND	ND
Virus	Group IV: +ssRNA	*Encephalopathy and retinopathy virus*	1 (0.02)	1 (0.05)	0 (0)	ND	ND	ND	5 (0.09)	3 (0.12)	2 (0.07)
Virus	Group IV: +ssRNA	*Cutthroat Trout virus*	2 (0.04)	2 (0.10)	0 (0)	1 (0.04)	1 (0.07)	0 (0)	7 (0.13)	7 (0.27)	0 (0)
Virus	Group IV: +ssRNA	*Putative Narna-like virus*	-	-	-	5 (0.21)	2 (0.13)	3 (0.33)	13 (0.25)	8 (0.31)	5 (0.19)
Virus	Group IV: +ssRNA	*Atlantic salmon Calicivirus*	ND	ND	ND	1 (0.04)	1 (0.07)	0 (0)	2 (0.04)	2 (0.08)	0 (0)
Virus	Group V: -ssRNA	*Salmon Pescarenavirus-2.*	ND	ND	ND	ND	ND	ND	1 (0.02)	1 (0.04)	0 (0)

*In parentheses is the proportion of sites within that particular group at which each pathogen species was detected. Cells containing "ND" represent pathogen species that were not detected in that year. Cells containing a dash represent pathogens that were not assessed in samples from that sampling season.

Supplementary Table 5. Viral detections segregated by the sample fraction and sampling year.

Group	Virus	*2016*			*2017*			*2018*		
		**Total**	**Cell**	**Free**	**Total**	**Cell**	**Free**	**Total**	**Cell**	**Free**
Group I: dsDNA	*Erythrocytic necrosis virus*	15	15	NA	12	12	2	26	26	3
Group III: dsRNA	*Piscine orthoreovirus*	ND	NA	NA	1	1	0	ND	ND	ND
Group IV: +ssRNA	*Encephalopathy and retinopathy virus*	1	NA	1	ND	ND	ND	5	0	5
Group IV: +ssRNA	*Cutthroat Trout virus*	2	NA	2	1	1	0	7	2	6
Group IV: +ssRNA	*Putative Narna-like virus*	ND	NA	NA	5	5	0	13	13	0
Group IV: +ssRNA	*Atlantic salmon Calicivirus*	ND	NA	NA	1	0	1	2	0	2
Group V: -ssRNA	*Salmon Pescarenavirus-2*	ND	NA	NA	ND	ND	ND	1	1	0

*Cell (cell-associated) viral detections indicate the number of viral detections that occurred in the cellular (>0.22μm) sample fraction and Free (free-virus) detections represent the number of viral detections that occurred in the sub-cellular (<0.22 μm) sample fraction.
*In cases where a virus was detected in both cellular and sub-cellular fractions of a particular sample, the sum of Cell (cell-associated) and Free (free-virus) may be greater than the Total (total number of sites) for that species.

Supplementary Table 6. A Summary of fit of generalized linear mixed effects models from multi-year analysis.

*Model	Log Likelihood	**ΔAIC_C	***ω_c
Null Model	-720.4	32.1	0
Site Status	-704.1	6.2	0.044
Atlantic salmon eDNA	-701.0	0	0.956

*All models include a random effect on the intercept for sampling site as well as a random effect on slope and intercept for farm status and pathogen respectively. Multi-year models include a random effect for site nested within a year on the intercept and slope of the predictor. There was a random effect for species on the coefficient for each additional predictor variable to allow for variation among species in how the predictor affected the detection probability.

** The difference in AIC_c values, corrected for small sample sizes, between the specified model and the best model. ***The Akaike model weight for each specified model, indicates the likelihood of this model given the model fit calculated from AIC_C values.

Supplementary Table 7. Parameter estimates and standard error estimates from fitted GLMM's assessing the effect of site status (active or inactive) and Atlantic salmon DNA on exposure to surveyed pathogens in surrounding environments.

Model	β	SE	Odds Ratio	95% CI
Site Status	1.00	0.31	2.72	1.48, 5.02
Atlantic salmon eDNA	0.57	0.16	1.76	1.28, 2.42

*Odds ratios and 95% confidence intervals were calculated from beta coefficient and standard error estimates.

Supplementary Table 8. Summary statistics for temperature, salinity, and Secchi disc measurements across sampling sites and sampling years.

Year	Sites	Temperature Mean (SD)	Salinity Mean (SD)	Mean Secchi Depth	Mean Cell Filtration Volume	Mean Viral Filtration Volume
2016	All	8.81 (0.44)	28.74 (1.45)	11.28 (4.01)	10.10 (0.36)	9.09 (0.24)
2016	Active	8.78 (0.34)	29.08 (1.36)	11.26 (4.37)	10.18 (0.49)	9.1 (0.24)
2016	Inactive	8.83 (0.49)	28.55 (1.48)	11.29 (3.86)	10.05 (0.27)	9.08 (0.25)
2016	t-test (p-value)	**0.67**	**0.18**	**0.98**	**0.30**	**0.80**
2017	All	12.52 (1.83)	27.25 (6.30)	6.68 (3.68)	14.66 (1.82)	10.99 (2.19)
2017	Active	12.63 (1.88)	26.79 (6.60)	6.86 (4.14)	14.44 (1.92)	10.81 (2.60)
2017	Inactive	12.27 (1.81)	28.40 (5.86)	6.32 (2.78)	15.02 (1.70)	11.28 (1.34)
2017	t-test (p-value)	**0.69**	**0.60**	**0.73**	**0.45**	**0.57**
2018	All	12.80 (2.28)	26.28 (3.96)	7.62 (3.39)	11.36 (0.55)	9.37 (0.40)
2018	Active	12.52 (1.81)	26.27 (3.85)	7.36 (3.09)	11.30 (0.54)	9.33 (0.37)
2018	Inactive	13.05 (2.65)	26.28 (4.14)	7.85 (3.68)	11.41 (0.56)	9.40 (0.43)
2018	t-test (p-value)	**0.41**	**1.00**	**0.62**	**0.50**	**0.50**

*Below each metric, we report significance levels (p-values) of differences between active and inactive sites based on two sample T-tests.

Supplementary Table 9 Model results for GLM's assessing the association between microparasite detections and filtered seawater volume.

Year	Model	β	SE	p-value
2016	Cellular (> 0.22 μm) Volume	-0.06	0.11	0.61
	Viral (< 0.22 μm) Volume	0.02	1.00	0.99
2017	Cellular (> 0.22 μm) Volume	-0.004	0.12	0.97
	Viral (< 0.22 μm) Volume	0.2	0.25	0.43
2018	Cellular (> 0.22 μm) Volume	-0.02	0.07	0.74
	Viral (< 0.22 μm) Volume	0.02	0.15	0.90

* Cellular Volume refers to the volume of seawater processed during the first filtration and Viral Volume refers to the volume of flocculated seawater filtered during the second filtration step. *Models failed to converge with the full random-effect structure reported for other GLMMs in this study; therefore, values reported above were obtained from GLM models with centered filtration volume as a predictor variable and binomial microparasite detections as a response variable.

See FILENAME: "Shea_Supp_Data_File.xlsx"

Supplementary File 1 (Electronic Appendix)

A table containing qPCR cycle threshold (Ct) values, coordinates, temperature, salinity, and Secchi disc measurements for all sites sampled in 2016, 2017, and 2018, as well as site status and filtration volumes. Data for all microparasites are aggregated into a single file, resulting in a duplication of site metrics for each microparasite surveyed.

DOI: https://doi.org/10.5061/dryad.r7sqv9s98

Supplementary References

1. Holmes RW. 1970 the Secchi Disk in Turbid Coastal Waters. *Limnol. Oceanogr.* **15**, 688–694. (doi:10.4319/lo.1970.15.5.0688)
2. Griffiths R, Whiteley A, O'Donnell A. 2000 Rapid method for coextraction of DNA and RNA from natural environments for analysis of …. *Appl. Environ. Microbiol.* **66**, 5488–5491. (doi:10.1128/aem.66.12.5488-5491.2000)
3. Doyle JJ, Doyle JL. 1987 A Rapid DNA Isolation Procedure for Small Quantities of Fresh Leaf Tissue. *Phytochem. Bull.* **19**, 11–15.
4. Miller KM *et al.* 2016 Report on the Performance Evaluation of the Fluidigm BioMark Platform for High- Throughput Microbe Monitoring in Salmon. *DFO CSAS Rep.* **Research D**, 1–282. (doi:10.13140/RG.2.2.15360.84487)
5. Miller K. 2019 Unpublished Data.
6. Atkinson S, Carlsson JEL, Ball B, Egan D, Kelly-Quinn M, Whelan K, Carlsson J. 2018 A quantitative PCR-based environmental DNA assay for detecting Atlantic salmon (Salmo salar L.). *Aquat. Conserv. Mar. Freshw. Ecosyst.* **28**, 1238–1243. (doi:10.1002/aqc.2931)
7. Rasmussen Hellberg RS, Morrissey MT, Hanner RH. 2010 A Multiplex PCR method for the identification of commercially important salmon and trout species (Oncorhynchus and Salmo) in North America. *J. Food Sci.* **75**. (doi:10.1111/j.1750-3841.2010.01752.x)
8. White VC, Morado JF, Crosson LM, Vadopalas B, Friedman CS. 2013 Development and validation of a quantitative PCR assay for Ichthyophonus spp. *Dis. Aquat. Organ.* **104**, 69–81. (doi:10.3354/dao02579)
9. Fringuelli E, Gordon AW, Rodger H, Welsh MD, Graham DA. 2012 Detection of neoparamoeba perurans by duplex quantitative taqman real-time PCR in formalin-fixed, paraffin-embedded atlantic salmonid gill tissues. *J. Fish Dis.* **35**, 711–724. (doi:10.1111/j.1365-2761.2012.01395.x)
10. Funk VA, Raap M, Sojonky K, Jones S, Robinson J, Falkenberg C, Miller KM. 2007 Development and validation of an RNA- and DNA- based quantitative PCR assay for determination of Kudoa thyrsites infection levels in Atlantic salmon Salmo salar. **75**, 239–249. (doi:10.3354/dao075239)
11. Jørgensen A, Nylund A, Nikolaisen V, Alexandersen S, Karlsbakk E. 2011 Real-time PCR detection of Parvicapsula pseudobranchicola (Myxozoa: Myxosporea) in wild salmonids in Norway. *J. Fish Dis.* **34**, 365–371. (doi:10.1111/j.1365-2761.2011.01248.x)
12. Hallett SL, Bartholomew JL. 2009 Development and application of a duplex QPCR for river water samples to monitor the myxozoan parasite Parvicapsula minibicornis. *Dis. Aquat. Organ.* **86**, 39–50. (doi:10.3354/dao02104)
13. Foltz JR, Plant KP, Overturf K, Clemens K, Powell MS. 2009 Detection of Nucleospora salmonis in steelhead trout, Oncorhynchus mykiss (Walbaum), using quantitative polymerase chain reaction (qPCR). *J. Fish Dis.* **32**, 551–555. (doi:10.1111/j.1365-2761.2009.00995.x)
14. Nylund S, Nylund A, Watanabe K, Arnesen CE, Karlsbakk E. 2010 Paranucleospora theridion n. gen., n. sp. (Microsporidia, Enterocytozoonidae) with a Life Cycle in the Salmon Louse (Lepeophtheirus salmonis, Copepoda) and Atlantic Salmon (Salmo salar). *J. Eukaryot. Microbiol.* **57**, 95–114. (doi:10.1111/j.1550-7408.2009.00451.x)
15. Duesund H, Nylund S, Watanabe K, Ottem KF, Nylund A. 2010 Characterization of a

VHS virus genotype III isolated from rainbow trout (Oncorhychus mykiss) at a marine site on the west coast of Norway. *Virol. J.* **7**, 1–15. (doi:10.1186/1743-422X-7-19)

16. Fringuelli E, Savage PD, Gordon A, Baxter EJ, Rodger HD, Graham DA. 2012 Development of a quantitative real-time PCR for the detection of Tenacibaculum maritimum and its application to field samples. *J. Fish Dis.* **35**, 579–590. (doi:10.1111/j.1365-2761.2012.01377.x)
17. Keeling SE, Johnston C, Wallis R, Brosnahan CL, Gudkovs N, McDonald WL. 2012 Development and validation of real-time PCR for the detection of Yersinia ruckeri. *J. Fish Dis.* **35**, 119–125. (doi:10.1111/j.1365-2761.2011.01327.x)
18. Nylund A, Watanabe K, Nylund S, Karlsen M, Sæther PA, Arnesen CE, Karlsbakk E. 2008 Morphogenesis of salmonid gill poxvirus associated with proliferative gill disease in farmed Atlantic salmon (Salmo salar) in Norway. *Arch. Virol.* **153**, 1299–1309. (doi:10.1007/s00705-008-0117-7)
19. Duesund H, Nylund S, Watanabe K, Ottem KF, Nylund A. 2010 Characterization of a VHS virus genotype III isolated from rainbow trout (Oncorhychus mykiss) at a marine site on the west coast of Norway. *Virol. J.* **7**, 19. (doi:10.1186/1743-422X-7-19)
20. Keeling SE, Brosnahan CL, Johnston C, Wallis R, Gudkovs N, Mcdonald WL. 2013 Development and validation of a real-time PCR assay for the detection of Aeromonas salmonicida. *J. Fish Dis.* **36**, 495–503. (doi:10.1111/jfd.12014)
21. Corbeil S, McColl KA, Crane MSJ. 2003 Development of a TaqMan quantitative PCR assay for the identification of Piscirickettsia salmonis. *Bull. Eur. Assoc. Fish Pathol.* **23**, 95–101.
22. Mitchell SO, Steinum TM, Toenshoff ER, Kvellestad A, Falk K, Horn M, Colquhoun DJ. 2013 Candidatus branchiomonas cysticola is a common agent of epitheliocysts in seawater-farmed atlantic salmon salmo salar in norway and ireland. *Dis. Aquat. Organ.* **103**, 35–43. (doi:10.3354/dao02563)
23. Powell M, Overturf K, Hogge C, Johnson K. 2005 Detection of Renibacterium salmoninarum in chinook salmon, Oncorhynchus tshawytscha (Walbaum), using quantitative PCR. *J. Fish Dis.* **28**, 615–622. (doi:10.1111/j.1365-2761.2005.00669.x)
24. Purcell MK, Pearman-Gillman S, Thompson RL, Gregg JL, Hart LM, Winton JR, Emmenegger EJ, Hershberger PK. 2016 Identification of the major capsid protein of erythrocytic necrosis virus (ENV) and development of quantitative real-time PCR assays for quantification of ENV DNA. *J. Vet. Diagnostic Investig.* **28**, 382–391. (doi:10.1177/1040638716646411)
25. Wiik-Nielsen CR, Ski PMR, Aunsmo A, Løvoll M. 2012 Prevalence of viral RNA from piscine reovirus and piscine myocarditis virus in Atlantic salmon, Salmo salar L., broodfish and progeny. *J. Fish Dis.* **35**, 169–171. (doi:10.1111/j.1365-2761.2011.01328.x)
26. Mordecai GJ *et al.* 2020 Emerging viruses in British Columbia Salmon discovered via a viral immune response biomarker panel and metatranscriptomic sequencing. *bioRxiv* (doi:10.1101/2020.02.13.948026)
27. Mordecai GJ *et al.* 2019 Endangered wild salmon infected by newly discovered viruses. *Elife* **8**, 1–18. (doi:10.7554/elife.47615)
28. Korsnes K, Devold M, Nerland AH, Nylund A. 2005 Viral encephalopathy and retinopathy (VER) in Atlantic salmon Salmo salar after intraperitoneal challenge with a nodavirus from Atlantic halibut Hippoglossus hippoglossus. *Dis. Aquat. Organ.* **68**, 7–15. (doi:10.3354/dao068007)
